# Oxidative-Stress-Associated Molecular Signatures in Immune-Mediated Diseases: A Systematic Review Integrating Machine Learning and Systems Biology Approaches

**DOI:** 10.3390/antiox15050548

**Published:** 2026-04-26

**Authors:** Rahul Mittal, Eavin A. Valerio, Vedaant Mutha, Aaryan Raj, Khemraj Hirani

**Affiliations:** 1 Diabetes Research Institute, University of Miami Miller School of Medicine, Miami, FL 33136, USA; evalerio@ufl.edu (E.A.V.); vmutha@ufl.edu (V.M.); aaryanraj@ufl.edu (A.R.); 2 Division of Endocrinology, Diabetes, and Metabolism, Department of Medicine, University of Miami Miller School of Medicine, Miami, FL 33136, USA; 3 University of Florida, Gainesville, FL 32611, USA

**Keywords:** oxidative stress, immune-mediated diseases, machine learning, bioinformatics, systems biology, multi-omics, biomarkers

## Abstract

Oxidative stress is a key contributor to the pathogenesis of immune-mediated diseases through its effects on cellular metabolism, mitochondrial function, immune signaling pathways, and inflammatory tissue injury. Disruption of redox homeostasis promotes metabolic reprogramming and persistent activation of innate and adaptive immune responses, contributing to disease progression across multiple inflammatory and autoimmune disorders. Recent advances in high throughput molecular technologies have generated large scale multi-omics datasets that enable comprehensive investigation of redox-associated mechanisms at a systems level. Integration of these datasets with computational analytical approaches has facilitated the identification of multidimensional molecular signatures associated with disease development and progression. This systematic review evaluates studies applying computational frameworks to analyze redox-related molecular data in immune-mediated diseases including multiple sclerosis, systemic lupus erythematosus, lupus nephritis, rheumatoid arthritis, Sjögren’s syndrome, and inflammatory bowel disease. Across the reviewed studies, oxidative stress associated with molecular signatures were consistently linked to immune activation, mitochondrial metabolism, and inflammatory signaling pathways. Computational analyses also identified regulatory genes involved in antioxidant defense and metabolic regulation, as well as pathways associated with regulated cell death. These findings highlight the translational potential of computational redox analysis for biomarker discovery, disease stratification, and development of targeted therapeutic strategies aimed at restoring redox balance and improving clinical management of immune-mediated diseases.

## 1. Introduction

Autoimmune diseases comprise a diverse group of chronic inflammatory disorders characterized by loss of immunological tolerance and the development of immune responses directed against self-antigens [1,2,3,4,5,6,7,8,9,10]. These conditions include multiple sclerosis (MS), systemic lupus erythematosus (SLE), rheumatoid arthritis (RA), Sjögren’s syndrome (SS), type 1 diabetes (T1D), and inflammatory bowel disease (IBD) [11,12,13,14,15,16]. Collectively, they affect multiple organ systems and represent a significant global health burden [17,18,19,20,21,22]. Although clinical manifestations and affected tissues differ among these disorders, many share common pathogenic mechanisms including persistent immune activation, dysregulated cytokine signaling, mitochondrial dysfunction, and alterations in cellular metabolism [23,24,25,26,27,28]. Among these interconnected processes, disruption of redox balance has emerged as a central factor contributing to immune dysregulation and inflammatory tissue injury [29,30]. Cellular redox homeostasis is maintained through complex interactions between reactive oxygen species (ROS), reactive nitrogen species (RNS), and endogenous antioxidant systems that include enzymatic components such as superoxide dismutases, catalase, and glutathione dependent pathways [31,32,33,34]. Under physiological conditions, ROSs function as signaling mediators that regulate intracellular pathways involved in immune cell activation, transcriptional regulation, and host defense mechanisms [35,36,37]. However, excessive ROS production or impairment of antioxidant defenses can disrupt this balance and result in oxidative stress [38,39,40]. This altered redox environment leads to molecular damage affecting lipids, proteins, and nucleic acids while simultaneously amplifying inflammatory signaling pathways that sustain immune-mediated tissue injury [41,42,43].

**Figure 1:**
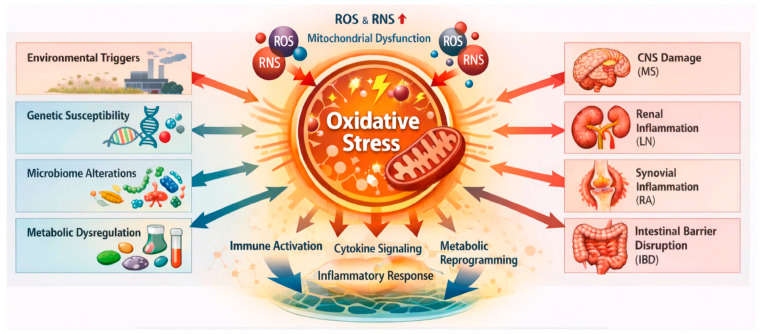
Oxidative stress-centered framework linking environmental and molecular triggers to immune-mediated disease pathology. Environmental exposures, genetic susceptibility, microbiome alterations, and metabolic dysregulation disrupt cellular redox homeostasis, leading to increased production of reactive oxygen species (ROSs) and reactive nitrogen species (RNSs) and mitochondrial dysfunction. This redox imbalance promotes immune activation, cytokine signaling, inflammatory responses, and metabolic reprogramming. Sustained oxidative stress-driven signaling contributes to tissue-specific pathology across immune-mediated diseases, including central nervous system damage in multiple sclerosis (MS), renal inflammation in lupus nephritis (LN), synovial inflammation in rheumatoid arthritis (RA), and intestinal barrier disruption in inflammatory bowel disease (IBD). Created in BioRender. Mittal, R. (2026) https://BioRender.com/yucv2gp (accessed on 19 April 2026).

The pathogenic contribution of oxidative stress to immune-mediated disorders has been demonstrated across multiple biological systems and disease contexts [44,45,46,47]. In neuroinflammatory conditions such as MS, increased ROS production contributes to mitochondrial dysfunction, demyelination, and axonal degeneration within the central nervous system [48,49,50]. Similarly, in SLE and lupus nephritis (LN), oxidative stress promotes immune complex formation, endothelial dysfunction, and inflammatory injury within renal tissue [46,51,52]. In RA, oxidative stress contributes to synovial hyperplasia, activation of fibroblast like synoviocytes, and increased production of inflammatory mediators that drive cartilage and bone destruction [53,54,55]. Within the gastrointestinal tract, oxidative stress plays an important role in epithelial barrier disruption, microbial dysbiosis, and persistent mucosal inflammation observed in IBD [44,56]. Beyond direct cytotoxic effects, oxidative stress also influences immune cell differentiation and signaling by modulating intracellular pathways that regulate antigen presentation, T-cell activation, and cytokine production [57,58,59] (Figure 1). These processes involve complex regulatory interactions between metabolic pathways, transcriptional networks, and immune signaling cascades, which together suggest the multifaceted role of redox biology in immune-mediated disease pathogenesis [60,61,62] (Figure 1).

Recent advances in high throughput molecular technologies have provided new opportunities to investigate these mechanisms at a systems level [63,64]. Multi-omics approaches including transcriptomics, metabolomics, proteomics, and microbiome profiling enable comprehensive characterization of molecular alterations associated with disease development and progression [65,66,67]. Although these technologies generate large volumes of biological data capable of capturing the multidimensional nature of immune-mediated pathology, the complexity of these datasets requires advanced computational methods for meaningful interpretation. Consequently, computational frameworks including bioinformatics, machine learning, and systems biology have become essential tools for analyzing redox-associated molecular datasets [68,69,70]. Machine learning algorithms such as random forest (RF), support vector machine (SVM), and artificial neural networks (ANNs) have demonstrated considerable potential for identifying diagnostic biomarkers and classifying disease states based on complex molecular signatures derived from metabolomic and transcriptomic data [63,71,72,73]. In addition, network-based analytical approaches including Weighted Gene Co-expression Network Analysis (WGCNA) enable the reconstruction of molecular interaction networks that reveal regulatory hubs linking oxidative stress with immune signaling pathways and metabolic reprogramming [74,75,76,77].

Integration of computational analysis with redox biology has begun to provide important insight into the mechanisms underlying immune-mediated diseases [78,79,80,81]. Computational analysis of metabolomic datasets has identified metabolic signatures associated with oxidative stress that distinguish affected individuals from healthy controls with high diagnostic accuracy [82]. Transcriptomic network analyses have revealed regulatory genes and signaling pathways that connect oxidative stress with immune activation, mitochondrial metabolism, and inflammatory signaling [83,84,85]. In addition, emerging evidence indicates that oxidative stress contributes to previously underrecognized mechanisms of tissue injury including regulated cell death pathways such as ferroptosis and cuproptosis, which involve lipid peroxidation and metal-dependent oxidative damage [86,87,88,89]. These pathways represent important links between metabolic dysregulation and immune-mediated tissue injury and further highlight the complex role of redox processes in inflammatory disease mechanisms [90,91].

**Figure 2:**
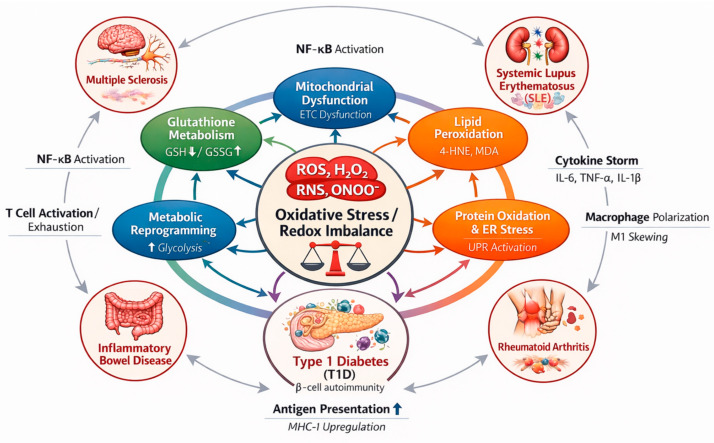
A unified redox network links shared oxidative stress pathways across autoimmune and inflammatory diseases. A schematic representation of a unified redox framework in which increased reactive oxygen and nitrogen species (ROSs/RNSs) drives interconnected pathways including glutathione dysregulation, mitochondrial dysfunction, lipid peroxidation, protein oxidation/ER stress, DNA damage, and metabolic reprogramming. These processes collectively promote NF-κB activation, pro-inflammatory cytokine production, immune cell activation, and enhanced antigen presentation. Despite distinct tissue-specific manifestations, these shared mechanisms contribute to pathogenesis across multiple sclerosis (MS), systemic lupus erythematosus (SLE), rheumatoid arthritis (RA), inflammatory bowel disease (IBD), and type 1 diabetes (T1D). Created in BioRender. Mittal, R. (2026) https://BioRender.com/bj3x8h2 (accessed on 19 April 2026). Abbreviations: ROS, reactive oxygen species; RNS, reactive nitrogen species; NF-κB, nuclear factor kappa B; IL, interleukin; TNF-α, tumor necrosis factor alpha; MHC-I, major histocompatibility complex class I; GSH, reduced glutathione; GSSG, oxidized glutathione; ETC, electron transport chain; ER, endoplasmic reticulum; UPR, unfolded protein response; 4-HNE, 4-hydroxynonenal; MDA, malondialdehyde; MS, multiple sclerosis; SLE, systemic lupus erythematosus; RA, rheumatoid arthritis; IBD, inflammatory bowel disease; T1D, type 1 diabetes.

Despite increasing recognition of the importance of oxidative stress in immune-mediated diseases, the rapidly expanding body of literature integrating computational analysis with redox biology remains limited. Individual studies frequently focus on specific diseases, datasets, or analytical methods, which limit the ability to identify shared molecular patterns across different disorders. A systematic synthesis of studies employing computational approaches to investigate redox-associated mechanisms is therefore necessary in order to evaluate how these analytical frameworks are currently applied and to determine their potential relevance for biomarker discovery and mechanistic understanding. Furthermore, assessment of whether redox-associated molecular signatures are consistent across different diseases may provide insight into common pathways underlying immune dysregulation and inflammatory tissue injury (Figure 2).

The objective of this systematic review is to evaluate recent investigations that apply computational analytical approaches to study redox-associated mechanisms in immune-mediated diseases. By synthesizing evidence from studies integrating machine learning, bioinformatics, and systems biology with multi-omics datasets, this review aims to identify recurrent molecular signatures, regulatory networks, and metabolic pathways associated with oxidative stress in inflammatory pathology. In addition, this review evaluates the potential translational relevance of computational redox analysis for biomarker discovery, disease classification, and therapeutic targeting. Through a systematic evaluation of current evidence, this work seeks to provide a comprehensive overview of how computational methodologies are advancing the understanding of oxidative-stress-related mechanisms in immune-mediated diseases.

While previous reviews have examined oxidative stress and redox biology in immune-mediated diseases or have evaluated computational and ML approaches for biomarker discovery, these domains have largely been addressed in isolation. The present study provides a systematic and integrative synthesis of investigations that combine multi-omics datasets with computational analytical frameworks to interrogate OS-associated molecular mechanisms across multiple immune-mediated diseases. By comparatively evaluating metabolomic, transcriptomic, and multi-omics studies, this review identifies convergent redox-associated pathways and examines the consistency, reproducibility, and biological relevance of computationally derived molecular signatures. In addition, this work incorporates a critical assessment of ML-based methodologies, with emphasis on factors influencing model robustness, generalizability, and interpretability. Importantly, this review adopts a cross-disease perspective to delineate shared mechanistic features of redox dysregulation, rather than focusing on single-disease contexts. This integrated approach provides a conceptual and methodological framework for understanding how computational analyses are advancing the study of redox biology in immune-mediated diseases and highlights key challenges that must be addressed to facilitate translation into clinically applicable biomarkers.

## 2. Materials and Methods

### 2.1. Study Design

This systematic review adhered to the PRISMA (Preferred Reporting Items for Systematic Reviews and Meta Analyses) guidelines to ensure methodological rigor and transparency. A protocol for this systematic review was developed a priori and subsequently registered in the INPLASY database (INPLASY202630051) prior to commencement of the study.

The objective of this review was to systematically evaluate studies investigating oxidative-stress-associated mechanisms in immune-mediated diseases using computational analytical approaches. The review focused on studies applying computational techniques including machine learning, bioinformatics, or systems biology to analyze molecular datasets associated with oxidative stress in autoimmune diseases.

### 2.2. Literature Search Strategy

A comprehensive literature search was conducted to identify studies investigating oxidative-stress-associated molecular mechanisms in MS, SLE, LN, RA, SS, and IBD using computational analytical approaches. Multiple electronic databases, including PubMed, Scopus, Web of Science, and Embase, were systematically searched to ensure broad biomedical coverage. Google Scholar was used as a supplementary source to capture relevant studies not indexed in primary databases. The search strategy incorporated both free text keywords and controlled vocabulary terms to maximize the sensitivity and specificity of the literature retrieval process. Keywords included combinations of the terms such as oxidative stress, redox biology, reactive oxygen species, machine learning, bioinformatics, computational analysis, metabolomics, transcriptomics, systems biology, biomarker discovery, MS, SLE, LN, RA, SS, and IBD. Boolean operators were used to combine search terms and expand the search scope such as (“Oxidative Stress” OR “Reactive Oxygen Species” OR “redox biology”) AND (“Machine Learning” OR “bioinformatics” OR “computational analysis” OR “systems biology”) AND (“Multiple Sclerosis” OR “Systemic Lupus Erythematosus” OR “Lupus Nephritis” OR “Rheumatoid Arthritis” OR “Sjögren’s Syndrome” OR “Inflammatory Bowel Disease”) AND (“metabolomics” OR “transcriptomics” OR “proteomics” OR “multi-omics”).

Medical Subject Headings were also incorporated to improve search completeness. The MeSH terms included Oxidative Stress, Reactive Oxygen Species, Machine Learning, Bioinformatics, Systems Biology, Metabolomics, Transcriptome, Biomarkers, Multiple Sclerosis, Systemic Lupus Erythematosus, Lupus Nephritis, Rheumatoid Arthritis, Sjögren’s Syndrome, and Inflammatory Bowel Diseases.

### 2.3. Study Screening Process

Study selection was conducted using a multistage screening process. Titles and abstracts of all retrieved records were initially screened to remove duplicate publications and clearly irrelevant studies. Articles that did not investigate oxidative-stress-related mechanisms, computational analysis, or the diseases of interest including MS, SLE, LN, RA, SS, or IBD were excluded during this stage. Two independent reviewers (EV and VM) screened all titles and abstracts for eligibility. Discrepancies between reviewers were resolved through discussion or consultation with senior authors (RM and KH). Full text articles of potentially relevant studies were subsequently evaluated according to predefined inclusion and exclusion criteria. Only studies meeting these criteria were included in the final analysis.

### 2.4. Inclusion and Exclusion Criteria

Studies were included if they met the following criteria. First, the study investigated oxidative stress, redox biology, or reactive oxygen species related mechanisms in MS, SLE, LN, RA, SS, or IBD. Second, the study applied computational analytical approaches including machine learning algorithms, bioinformatic analysis, or systems biology methods. Third, the study utilized omics-based datasets including transcriptomics, metabolomics, proteomics, microbiome data, or integrated multi-omics approaches. Fourth, the study reported molecular or computational findings associated with oxidative-stress-related pathways.

Studies were excluded if they focused on diseases outside the scope of this review, did not apply computational analytical methods, or lacked molecular data related to oxidative stress. Review articles, conference abstracts, editorials, and studies without primary data were also excluded.

### 2.5. Data Extraction

Data extraction was performed for all studies that met the inclusion criteria. Information collected from each study included author name, year of publication, disease type such as MS, SLE, LN, RA, SS, or IBD, dataset type, computational methodology, and key findings related to oxidative-stress-associated molecular mechanisms. Additional information regarding identified biomarkers, metabolic pathways, gene networks, and immune signaling pathways was also recorded. All data were extracted by at least two trained reviewers (EV and VM) to ensure accuracy and consistency. Any discrepancies in extracted data were resolved through discussion among the reviewers or consultation with senior authors (RM and KH)

### 2.6. Computational Approaches Evaluated

The included studies applied a variety of computational techniques to analyze molecular datasets associated with oxidative stress. Machine learning algorithms including RF, SVM, ANN, and gradient boosting models were frequently used to identify diagnostic biomarkers and classify disease states based on molecular signatures. Network-based analytical approaches such as WGCNA were also applied to identify regulatory gene networks associated with immune activation and redox dysregulation. Integration of multi-omics datasets enabled identification of complex molecular interactions linking metabolic pathways, transcriptional regulation, and immune signaling processes.

### 2.7. Data Synthesis

Due to heterogeneity in study design, dataset type, and computational methodology among the included studies, quantitative meta-analysis was not performed. Instead, findings were synthesized qualitatively through comparative evaluation of molecular signatures, regulatory pathways, and computational models associated with oxidative stress in MS, SLE, LN, RA, SS, and IBD. This narrative synthesis approach enabled identification of recurring molecular patterns and computational strategies contributing to the understanding of redox biology in inflammatory disease contexts.

### 2.8. Risk of Bias Assessment

Risk of bias for the included studies was assessed using the Prediction Model Risk of Bias Assessment Tool (PROBAST). This tool evaluates potential methodological bias in studies involving predictive models and machine-learning-based analyses. Four domains were evaluated for each study. The first domain assessed bias related to participants and data sources, including cohort selection and dataset representativeness. The second domain evaluated predictors, specifically the definition and measurement of molecular variables such as transcriptomic, metabolomic, proteomic, or multi-omics features used as model inputs. The third domain evaluated outcome definitions, including classification of disease states such as MS, SLE, LN, RA, SS, and IBD. The fourth domain assessed analysis and artificial intelligence model validation, including model development procedures, validation methods, potential overfitting, and reporting transparency. Each domain was classified as low risk, unclear risk, or high risk of bias.

## 3. Results

### 3.1. Study Selection

**Figure 3:**
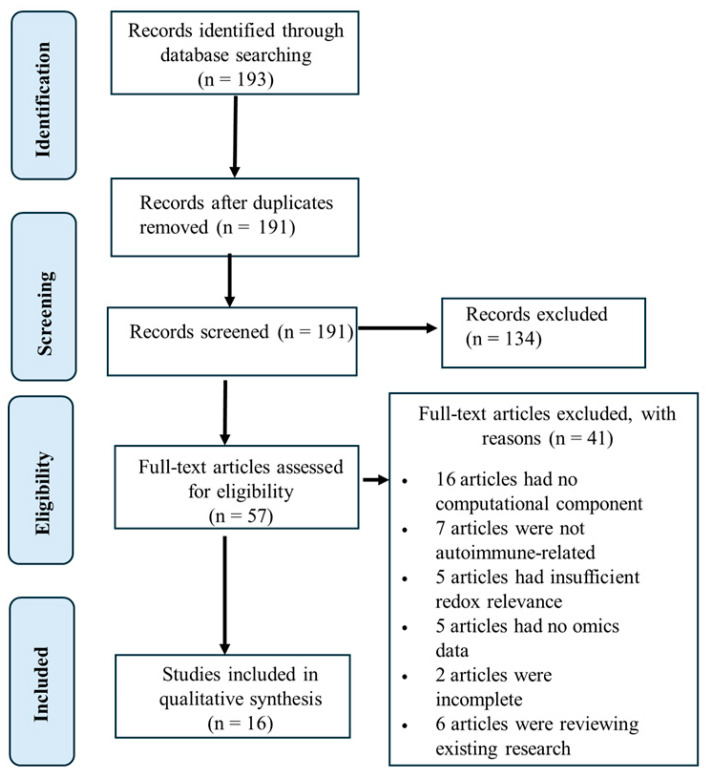
PRISMA (Preferred Reporting Items for Systematic Reviews and Meta-Analyses) flow diagram showing the study selection process for identifying and screening studies included in the systematic review.

The literature search initially identified 193 records through database searching (Figure 3). Following removal of duplicate entries, 191 records were retained for title and abstract screening. Of these, 134 records were excluded based on lack of relevance to the study objectives. Fifty-seven full text articles were subsequently assessed for eligibility. Forty-one studies were excluded at the full text stage due to absence of computational analytical components, lack of relevance to autoimmune or inflammatory diseases, insufficient focus on redox-associated mechanisms, absence of omics-based datasets, incomplete reporting of study data, or classification as review articles. Following the application of the predefined inclusion criteria, 16 studies were included in the final qualitative synthesis. The key characteristics of the included studies are summarized in Table 1. To enable systematic cross-study comparison, detailed methodological and analytical features are further presented in Table 2 and Supplementary Table S1. Comparative evaluation indicates that the majority of studies employed internal validation strategies, with relatively few incorporating independent external cohorts, thereby raising concerns regarding model generalizability and robustness. Metabolomic datasets were most frequently utilized, particularly in studies of inflammatory bowel disease and rheumatoid arthritis, whereas transcriptomic and integrative multi-omics approaches were more commonly applied in systemic lupus erythematosus and multiple sclerosis. Despite substantial methodological heterogeneity, several oxidative-stress-associated pathways were consistently identified across studies, including mitochondrial dysfunction, lipid peroxidation, glutathione metabolism, and redox-regulated inflammatory signaling. Collectively, these convergent findings support the existence of shared redox dysregulation mechanisms underlying the pathogenesis of immune-mediated diseases (Figure 2).

**Figure 4:**
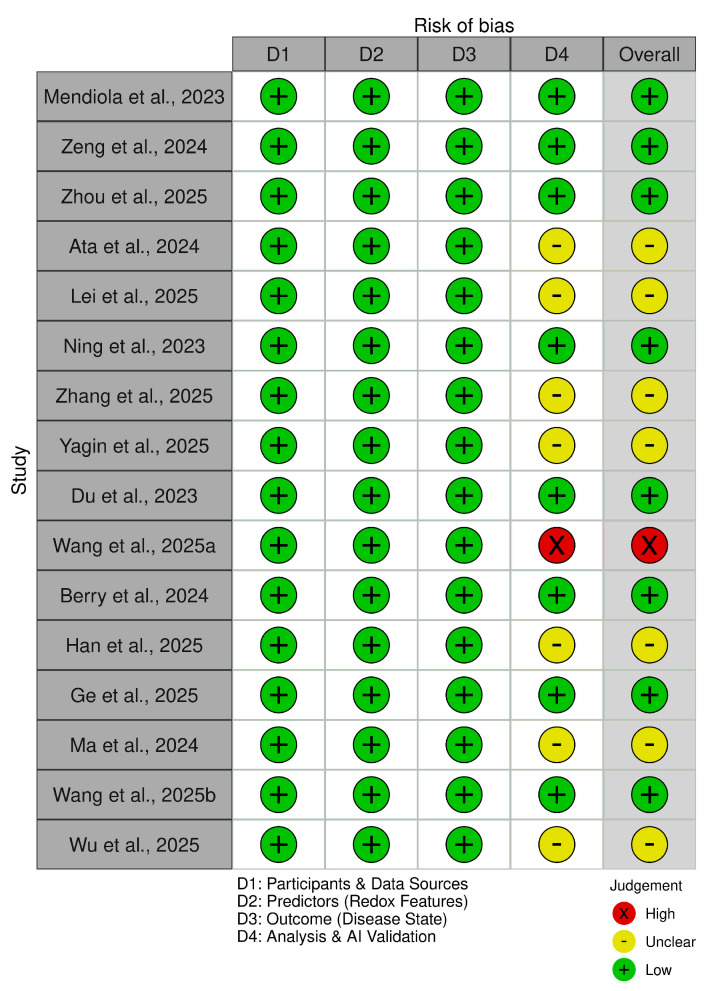
Risk of bias assessment of included studies using the Prediction Model Risk of Bias Assessment Tool (PROBAST). Green indicates low risk, yellow indicates unclear risk, and red indicates high risk of bias across the evaluated domains [76,78,79,82,83,92,93,94,95,96,97,98,99,100,101,102].

Risk of bias assessment indicated generally low methodological bias across the domains evaluating participants and data sources, predictors, and disease outcome definitions (Figure 4). Several studies demonstrated some concerns within the analytical domain, primarily related to limited external validation or incomplete reporting of model validation procedures. One study demonstrated high risk of bias within the analysis and artificial intelligence validation domain due to insufficient validation of the predictive model. Overall, the majority of included studies were classified as having low to moderate risk of bias, supporting the overall methodological robustness of the reviewed evidence.

### 3.2. Machine Learning Identification of Redox-Associated Metabolic Biomarkers

Several studies demonstrated that metabolomic signatures associated with oxidative stress provide substantial discriminatory power for AD classification when integrated with ML frameworks [92,93,94]. These approaches utilize high dimensional metabolomic datasets to identify patterns of biochemical dysregulation reflecting altered redox homeostasis and immune-driven metabolic reprogramming. Collectively, the studies analyzed in this review highlight the increasing role of computational modeling in identifying metabolite-based biomarkers capable of improving diagnostic accuracy and disease stratification across multiple ADs.

Ata et al. (2024) investigated the application of ANN for diagnosing MS using blood-based metabolomics data derived from a cohort of 756 individuals, including 515 MS patients and 241 HC [92]. Following data normalization and partitioning into training, validation, and testing sets, the authors constructed a feed forward ANN capable of capturing nonlinear relationships between metabolite concentrations and disease status. The model achieved an overall diagnostic accuracy of 87.0%, with a sensitivity of 82.5% and specificity of 89.0%, indicating strong predictive capability for distinguishing MS patients from HC. Importantly, the study demonstrated that metabolic profiles reflecting oxidative-stress-related pathways contributed substantially to classification performance. Metabolites associated with mitochondrial energy metabolism and redox balance were among the most informative predictive variables, suggesting that systemic metabolic disturbances linked to oxidative stress play a significant role in MS pathophysiology [92]. These findings support the potential of ANN-based metabolomic analysis as a noninvasive diagnostic approach capable of detecting subtle metabolic perturbations that occur during early MS progression.

Comparable observations were reported in RA. Yagin et al. (2025) applied an explainable boosting machine model to plasma metabolomic datasets in order to identify disease-associated metabolic signatures while preserving model interpretability [93]. Using metabolomic profiles from RA patients and HC, the investigators implemented the Synthetic Minority Oversampling Technique to address dataset imbalance and compared several ML approaches. Among the tested models, the explainable boosting machine algorithm demonstrated the highest diagnostic performance, achieving an area under the receiver operating characteristic curve of 0.901 with a sensitivity of approximately 87.8%. Metabolomic analysis revealed significant disruptions in pathways associated with cellular energy metabolism and oxidative stress. In particular, concentrations of pyruvic acid and phenylalanine were significantly elevated in RA samples, whereas citrulline and carnitine levels were reduced. These metabolites are closely linked to mitochondrial function, glycolytic flux, and nitrogen metabolism, processes commonly altered under conditions of increased ROS production. Furthermore, the interpretable structure of the model revealed nonlinear risk thresholds for several metabolites, indicating that metabolic perturbations contribute to disease risk in a concentration dependent manner [93]. These results highlight the value of integrating metabolomics with explainable ML methods to identify biologically meaningful metabolic signatures associated with RA pathogenesis.

In addition to disease specific models, Du et al. (2023) demonstrated the broader potential of metabolomics-driven ML approaches for differentiating among multiple ADs simultaneously [94]. In this investigation, urine and serum samples from 267 participants, including patients with SLE, RA, SS, and AS, were analyzed using LC MS/MS. ML models including NB, RF, and neural network algorithms were applied to classify disease status based on metabolomic profiles. Among the evaluated models, urine-based NB classifiers demonstrated the highest diagnostic performance, achieving an accuracy of 98.9% and an area under the receiver operating characteristic curve of 1.000 for distinguishing AD patients from HC. Metabolic pathway analysis revealed widespread dysregulation in amino acid metabolism, lipid metabolism, and central carbon metabolism. Several metabolites associated with oxidative stress responses, including alterations in tryptophan metabolism and phosphatidylcholine levels, were identified as key predictive features [94]. These findings suggest that urine metabolomics provides a highly sensitive and noninvasive diagnostic platform and that ML algorithms can effectively integrate complex metabolic signatures to discriminate among distinct AD subtypes.

### 3.3. Metabolomic and Microbiome Signatures in Inflammatory Bowel Disease

Increasing evidence indicates that metabolic dysregulation associated with oxidative stress and host–microbiome interactions play a central role in the pathogenesis of IBD. Recent investigations have combined metabolomics with ML approaches to identify diagnostic biomarkers and characterize metabolic pathways disrupted during intestinal inflammation [95,96]. These computational strategies enable the identification of disease-associated metabolic patterns by integrating high-dimensional metabolomic datasets with predictive modeling algorithms capable of capturing complex nonlinear biological relationships.

Lei et al. (2025) performed targeted metabolomic profiling of urinary metabolites to investigate alterations in central carbon metabolism in patients with UC and CD [95]. Using UHPLC–MS/MS, 49 metabolites associated with glycolysis and the TCA cycle were quantified in urine samples collected from UC patients, CD patients, and HC. ML models were subsequently developed to evaluate the diagnostic performance of these metabolic features. Among the algorithms tested, the RF model demonstrated optimal performance for UC classification with an AUC of 0.84, whereas the SVM model showed superior performance for CD diagnosis with an AUC of 0.93. Several metabolites, including xylose, L-fucose, and GlcNAc, were identified as key predictive features [95]. These metabolites are strongly associated with microbial carbohydrate metabolism and intestinal epithelial barrier integrity, suggesting that metabolic perturbations associated with gut microbial activity contribute to disease development. Notably, the diagnostic performance of the ML models remained robust across varying disease activity states, indicating that metabolic signatures associated with oxidative stress and microbial dysbiosis represent stable indicators of disease presence.

Ge et al. (2025) applied an integrated metabolomics and ML framework to characterize metabolic signatures associated with disease extent and inflammatory activity in UC [97]. Serum metabolomic profiling was performed using high-resolution LC–MS, enabling detection of a broad spectrum of metabolites reflecting host metabolic and inflammatory processes. Following data preprocessing and normalization, computational feature selection methods were applied to identify metabolites with the highest discriminatory potential. Supervised ML algorithms, including RF and SVM classifiers, were subsequently implemented to evaluate the predictive performance of selected metabolic features for distinguishing UC patients from HC and for stratifying disease severity. The resulting models demonstrated strong classification performance and identified a subset of metabolites contributing substantially to predictive accuracy. Pathway enrichment analysis revealed significant perturbations in metabolic pathways associated with oxidative stress and inflammatory metabolic reprogramming, including alterations in tryptophan–kynurenine metabolism, lipid-peroxidation-related lipid species, and metabolites involved in mitochondrial energy metabolism [97]. Dysregulation of these pathways is consistent with increased ROS generation and mitochondrial dysfunction within intestinal epithelial and immune cells during intestinal inflammation, highlighting the contribution of oxidative- stress-associated metabolic remodeling to UC pathophysiology.

Consistent with these findings, additional metabolomics-driven ML analyses have further characterized metabolic perturbations associated with inflammatory activity and therapeutic response in UC. Han et al. (2025) conducted an untargeted metabolomics investigation combined with supervised ML modeling to identify metabolic biomarkers associated with disease activity and therapeutic response in UC [98]. Serum metabolite profiles were generated using LC–MS-based untargeted metabolomics, enabling comprehensive detection of metabolic perturbations associated with intestinal inflammation. Computational preprocessing and dimensionality reduction techniques were applied to the metabolomic dataset to identify candidate metabolites contributing to disease classification. Predictive models were subsequently constructed using ML algorithms to distinguish active disease from remission states and to evaluate metabolic signatures associated with treatment response. The resulting models demonstrated strong predictive capability and identified metabolic features significantly associated with inflammatory activity. Pathway enrichment analysis revealed substantial dysregulation of metabolic pathways linked to oxidative stress and mitochondrial metabolism, including alterations in amino acid metabolism, fatty acid β-oxidation, and TCA cycle intermediates. Several metabolites involved in redox homeostasis and inflammatory metabolic pathways contributed significantly to model performance [98]. These findings suggest that oxidative-stress-associated metabolic dysregulation represents a key component of UC pathogenesis and demonstrating the utility of integrating metabolomics with ML approaches to identify computational biomarkers of inflammatory disease activity.

Building upon these metabolomics-driven ML investigations, additional systems-level studies integrating multi-omics datasets have further elucidated the complex interactions between host metabolism, microbial ecology, and oxidative-stress-associated inflammatory pathways in IBD. Ning et al. (2023) conducted cross-cohort integration of metagenomic and metabolomic data obtained from nine metagenomic cohorts and four metabolomic cohorts encompassing more than 1300 individuals [96]. The analysis revealed substantial alterations in microbial composition in patients with IBD compared with HC. In total, 74 microbial species demonstrated significantly altered abundance, including depletion of beneficial commensal bacteria such as *Faecalibacterium prausnitzii* and enrichment of species associated with inflammatory responses [96]. Functional metagenomic analysis identified 162 differentially expressed KEGG orthology genes, several of which were associated with bacterial stress responses and host–microbial metabolic exchange.

Integration of microbial features with metabolomic profiles enabled construction of an RF diagnostic model capable of distinguishing IBD patients from HC with high predictive accuracy, achieving an AUC of 0.98 in the Renji cohort and 0.93 in an independent validation cohort [96]. Metabolomic analysis identified 79 shared metabolites associated with IBD pathology, of which 13 metabolites were selected as core diagnostic biomarkers. Many of these metabolites were linked to amino acid metabolism, microbial fermentation pathways, and oxidative-stress-related metabolic processes. Multi-omics biological correlation analysis further revealed functional relationships between microbial species and host metabolic pathways, including disruptions in aminoacyl-tRNA biosynthesis and microbial biotransformation mechanisms [96]. These findings indicate that microbial metabolic activity substantially influences host redox balance and immune regulation within the intestinal microenvironment.

### 3.4. Oxidative Stress Gene Signatures in SLE and LN

Studies have identified gene expression signatures associated with oxidative stress that contribute to the molecular pathology of SLE and LN [78,82]. Through the integration of transcriptomic datasets with ML and network-based analytical methods, these investigations identified key regulatory genes linking redox imbalance with immune activation and tissue inflammation. Collectively, these findings demonstrate that oxidative-stress-related transcriptional programs play a significant role in shaping immune dysregulation and disease progression in lupus-associated disorders.

Zeng et al. (2024) investigated oxidative-stress-associated gene networks in LN using transcriptomic datasets derived from renal biopsy samples [82]. Through application of WGCNA, the authors identified a gene module significantly correlated with disease pathology. Subsequent feature selection using LASSO regression revealed four hub genes including *STAT1*, *PRODH*, *TXN2*, and *SETX*. Expression analysis demonstrated that *STAT1* and *SETX* were significantly upregulated in LN patients, whereas *PRODH* and *TXN2* were downregulated compared with HC. Functional enrichment analysis revealed that these genes were primarily associated with signaling pathways involved in immune activation and cellular stress responses, including the JAK–STAT and PI3K–Akt pathways. A predictive model constructed using these four genes demonstrated strong diagnostic performance with an AUC of 0.929 in validation datasets. Immune infiltration analysis further revealed increased infiltration of activated B-cells and CD8^+^ T-cells in LN tissue alongside decreased NK-cell abundance, suggesting that oxidative-stress-related transcriptional programs contribute to both immune activation and alterations in the renal immune microenvironment.

Complementary findings were reported by Zhou et al. (2025), who conducted a multi-omics analysis integrating transcriptomic datasets with serum metabolomic profiling to identify oxidative-stress-related biomarkers in SLE [78]. Through GSVA and ML-based feature selection, the investigators identified six hub genes associated with oxidative stress and mitochondrial metabolism including *ABCB1*, *AKR1C3*, *EIF2AK2*, *IFIH1*, *NPC1*, and *SCO2*. Validation analysis confirmed that *ABCB1*, *AKR1C3*, and *NPC1* were significantly downregulated in PBMCs from SLE patients, whereas *EIF2AK2*, *IFIH1*, and *SCO2* were significantly upregulated. Among these biomarkers, *SCO2* and *EIF2AK2* demonstrated particularly strong diagnostic performance with AUC values of 0.979 and 0.960, respectively. Metabolomic profiling further revealed significant alterations in metabolic pathways associated with energy metabolism and mitochondrial respiration, including oxidative phosphorylation and the TCA cycle [78]. These metabolic perturbations were accompanied by increased systemic reactive oxygen species levels and reduced antioxidant capacity.

Single-cell transcriptomic analysis performed within the same study further demonstrated that oxidative-stress-related gene expression exhibited strong cell-type specificity. *ABCB1* and *AKR1C3* expressions were predominantly localized within NK-cells, which were significantly depleted in SLE samples [78]. In contrast, *IFIH1* and *SCO2* expressions were enriched in monocytes and macrophages, which were expanded in patient samples. These findings suggest that oxidative-stress-associated transcriptional programs contribute to immune cell population shifts and inflammatory activation during lupus pathogenesis.

### 3.5. Redox Regulatory Networks and Systems Biology in Autoimmune Disease

Alterations in redox regulatory networks have emerged as a central mechanism contributing to immune dysregulation across multiple autoimmune diseases. Redox homeostasis is tightly regulated through interconnected antioxidant pathways, metabolic signaling networks, and transcriptional regulators that collectively control cellular responses to reactive oxygen species. Disruption of these networks can lead to metabolic reprogramming of immune cells, enhanced inflammatory signaling, and sustained tissue damage. Recent studies integrating multi-omics datasets with computational approaches have begun to reveal how redox-associated regulatory networks contribute to autoimmune disease pathogenesis.

Wang et al. (2025a) investigated the role of glutathione metabolism in SLE using an integrated framework combining Mendelian randomization, single-cell RNA sequencing, and ML-based feature selection [83]. Analysis of serum metabolite datasets identified the glutathione pathway as significantly enriched among metabolites causally associated with disease risk. Single-cell transcriptomic analysis of PBMCs further revealed heterogeneity within monocyte populations based on glutathione metabolic activity. Monocytes exhibiting elevated glutathione pathway activity displayed increased expression of inflammatory signaling pathways and enhanced communication with T-cells and NK-cells [83]. These findings suggest that metabolic reprogramming associated with glutathione regulation contributes to immune activation and inflammatory signaling in SLE.

To identify regulatory genes associated with glutathione metabolism, ML algorithms including LASSO regression, CatBoost, XGBoost, and NGBoost were applied to transcriptomic datasets [83]. This approach identified several candidate genes associated with glutathione-dependent redox regulation, with *LAP3* emerging as the most significant contributor to diagnostic model performance. Expression of *LAP3* was significantly elevated in SLE samples and demonstrated strong predictive value for disease classification, achieving an AUC of 0.935 in the training dataset and maintaining robust performance in independent validation cohorts. Further analysis revealed that *LAP3* expression was predominantly localized within monocytes and was associated with enhanced immune signaling through the LGALS9–CD44 ligand–receptor interaction pathway [83]. This pathway has been implicated in immune cell activation and inflammatory signaling, suggesting that increased activity of the LAP3 protein may contribute to sustained inflammatory responses in SLE.

Complementary systems-level insight into redox regulatory networks was provided by Berry et al. (2024), who investigated molecular heterogeneity in SS using large-scale serum proteomics combined with computational network reconstruction [99]. Using O-link proteomic profiling, 454 circulating proteins were quantified from serum samples obtained from patients with primary SS. Network reconstruction using the ARACNE algorithm revealed distinct molecular endotypes corresponding to clinically defined symptom clusters. Patients with dryness-dominant disease exhibited strong activation of adaptive immune pathways characterized by increased interferon-associated chemokines and B- cell-stimulating cytokines. In contrast, patients with high symptom burden and pain-dominant disease exhibited increased expression of innate inflammatory mediators including IL-6 and IL-1α, along with elevated levels of proteins involved in oxidative stress responses and metabolic regulation such as the APE1/Ref-1 protein, TIGAR protein, and transcription factors encoded by *FOXO1* and *BACH1* [99]. Importantly, stratified reanalysis of a phase III clinical trial evaluating the IL-6 receptor inhibitor tocilizumab demonstrated improved fatigue outcomes specifically in patients belonging to the inflammatory high-symptom burden subtype [99]. These findings demonstrate how systems biology approaches can reveal biologically distinct disease subtypes and identify patient populations that may benefit from targeted therapeutic interventions.

### 3.6. Redox-Associated Cell Death Pathways in Lupus Nephritis

Recent investigations have highlighted the contribution of oxidative-stress-associated programmed cell death pathways to the pathogenesis of LN. In particular, emerging evidence suggests that ferroptosis and cuproptosis represent important mechanisms linking redox imbalance with immune-mediated tissue injury. These forms of regulated cell death are characterized by the accumulation of lipid peroxidation products, mitochondrial dysfunction, and disruption of cellular metal ion homeostasis. Integration of transcriptomic datasets with ML approaches has enabled the identification of key regulatory genes associated with these pathways and their potential role in LN progression.

Zhang et al. (2025) investigated the intersection between ferroptosis and cuproptosis in LN using transcriptomic datasets derived from public gene expression repositories [86]. Through differential expression analysis, 31 ferroptosis-related cuproptosis genes were identified as significantly dysregulated in LN samples. To determine the most relevant biomarkers, the investigators applied several ML algorithms including LASSO regression, RF analysis, and SVM recursive feature elimination. This multi-algorithm approach identified two hub genes, *JUN* and *ZFP36*, as key regulators associated with LN pathology. Expression analysis demonstrated that *JUN* and *ZFP36* were significantly downregulated in LN patient samples compared with HC. Diagnostic modeling based on these two genes demonstrated excellent predictive performance, achieving an AUC of 1.000 within the training dataset. Functional enrichment analysis suggested that these genes participate in regulatory pathways associated with oxidative stress responses, inflammatory signaling, and cellular metal ion metabolism. The results indicate that disruption of ferroptosis- and cuproptosis-related regulatory mechanisms may contribute to renal inflammation and tissue damage in LN.

Experimental validation further supported the involvement of oxidative-stress-driven cell death pathways in LN [86]. In vivo studies using MRL/lpr mouse models demonstrated increased expression of ferroptosis-associated markers including transferrin receptor protein 1 and the lipid peroxidation product 4-hydroxynonenal. In addition, expression of the cuproptosis-associated protein ferredoxin 1 was elevated in renal tissue. In vitro experiments using lipopolysaccharide-induced cellular models of LN further confirmed increased production of ROS and elevated levels of malondialdehyde, indicating enhanced lipid peroxidation and oxidative damage. Immune infiltration analysis also revealed a significant relationship between these hub genes and immune cell recruitment within the renal microenvironment [86]. Reduced expression of *JUN* and *ZFP36* was negatively correlated with infiltration of monocytes and M2 macrophages, suggesting that suppression of these regulatory genes may contribute to increased inflammatory cell accumulation in LN tissue [86]. These findings indicate that redox-associated cell death pathways may influence both tissue injury and immune cell dynamics during disease progression.

### 3.7. Redox Regulation of Immune Signaling in Autoimmune and Neuroinflammatory Disease

Redox-dependent signaling pathways play a central role in regulating immune activation and inflammatory responses in autoimmune and neuroinflammatory diseases. ROSs function not only as metabolic byproducts but also as signaling mediators capable of modulating transcriptional regulation, immune cell differentiation, and cytokine signaling pathways. Dysregulation of intracellular redox balance can therefore influence both innate and adaptive immune responses by altering gene expression programs and signaling networks associated with inflammatory activation. Recent advances in computational biology have enabled systematic investigation of these mechanisms through the integration of transcriptomic and multi-omics datasets with ML and bioinformatic analytical approaches, providing insight into how oxidative-stress-associated molecular pathways contribute to immune-mediated disease progression.

Ma et al. (2024) applied an integrated bioinformatic and ML framework to identify oxidative-stress-associated transcriptional signatures in MS using publicly available transcriptomic datasets derived from peripheral blood samples [100]. Differential expression analysis identified a subset of oxidative-stress-related genes that were significantly dysregulated in MS patients compared with HC. Protein–protein interaction network reconstruction and subsequent feature selection using ML algorithms identified several hub genes including *MMP9*, *NFKB1*, *NFKBIA*, and *SRC*. Functional enrichment analysis demonstrated that these genes participate in pathways associated with NF-κB-mediated inflammatory signaling, cytokine regulation, and leukocyte activation. Diagnostic modeling incorporating these hub genes demonstrated strong predictive performance for distinguishing MS patients from HC, indicating that oxidative-stress-associated transcriptional programs contribute substantially to disease classification. These findings illustrate how computational interrogation of transcriptomic datasets can identify redox-regulated gene networks that influence immune signaling pathways during MS pathogenesis.

Consistent with these observations, transcriptomic investigations integrating ML analysis have provided further insight into immune signaling mechanisms associated with neuroinflammation. Wu et al. (2025) performed RNA-seq analysis of CNS tissues obtained from experimental autoimmune encephalomyelitis models in order to characterize transcriptional programs associated with microglial activation during inflammatory disease progression [101]. Differential expression analysis followed by ML-based feature selection identified gene expression signatures associated with activated microglial states within inflammatory lesions. Pathway enrichment analysis revealed significant activation of innate immune signaling pathways including TLR signaling, cytokine-mediated inflammatory responses, and oxidative-stress-associated signaling cascades. Network-based analyses further demonstrated enrichment of genes involved in ROS-mediated inflammatory signaling and microglial immune activation. Several candidate biomarkers identified through ML analysis demonstrated strong predictive capability for distinguishing inflammatory microglial phenotypes [101]. These results highlight the utility of integrating RNA-seq datasets with computational modeling approaches to identify transcriptional programs linking oxidative stress with innate immune activation during neuroinflammatory disease.

Extending these observations, integrative multi-omics approaches have identified mitochondrial regulators of ROS production that contribute to susceptibility across multiple autoimmune diseases. Wang et al. (2025b) performed a comprehensive analysis integrating genome-wide association datasets with transcriptomic and single-cell RNA-seq data in order to investigate shared genetic mechanisms underlying autoimmune disease pathogenesis [102]. Computational integration of these datasets using bioinformatic feature selection methods identified *ROMO1* as a key regulatory gene associated with autoimmune disease susceptibility. *ROMO1* encodes a mitochondrial membrane protein that regulates intracellular ROS production and contributes to mitochondrial redox homeostasis. Expression analysis demonstrated that *ROMO1* was significantly dysregulated in immune cell populations involved in inflammatory responses, particularly monocyte subsets associated with innate immune activation. Functional enrichment analysis further revealed that *ROMO1* participates in pathways related to mitochondrial metabolism, oxidative stress regulation, and immune signaling [102]. These findings highlight the importance of mitochondrial ROS-generating pathways in shaping immune responses and illustrate how integrative multi-omics approaches can identify shared redox-associated molecular mechanisms across autoimmune diseases.

Additional systems-level insight into redox-associated inflammatory signaling within the CNS has been provided by multi-omics investigations examining interactions between circulating factors and resident immune cells. Mendiola et al. (2023) investigated how blood-derived proteins influence microglial activation using an integrated multi-omics framework combining transcriptomic and proteomic analyses [79]. Exposure of microglia to plasma proteins triggered transcriptional programs associated with oxidative stress responses and inflammatory signaling pathways. The study identified fibrinogen as a key circulating mediator capable of inducing microglial activation. Interaction between fibrinogen and the integrin receptor CD11b encoded by *ITGAM* activated intracellular signaling cascades involving MAP2K2 and ERK1/2 (*MAPK1*/*MAPK3*) and stimulated NADPH oxidase activity, resulting in increased ROS production. Genetic disruption of the fibrinogen–CD11b interaction significantly attenuated microglial inflammatory activation and reduced expression of disease-associated genes including *APOE* and *CST7*. These findings demonstrate how vascular-derived signals can initiate oxidative stress-dependent inflammatory pathways within the CNS and illustrate the value of multi-omics integration for identifying molecular mechanisms linking redox signaling with neuroinflammatory disease processes.

## 4. Integrated Synthesis and Comparative Evaluation of Evidence

Although individual studies provide important insights into oxidative-stress-associated molecular mechanisms in immune-mediated diseases, a comparative synthesis reveals both convergent findings and notable methodological and analytical limitations (Table 2 and Supplementary Table S1). While Table 2 summarizes study-level characteristics and key findings, Supplementary Table S1 provides an integrated cross-study comparison highlighting recurring patterns and methodological variability. Across studies, a consistent pattern emerges in which oxidative-stress-related signatures are linked to key biological processes, including mitochondrial dysfunction, inflammatory signaling, glutathione metabolism, and metabolic reprogramming. These pathways were identified across multiple diseases and data types, suggesting that redox imbalance represents a shared mechanistic axis underlying immune dysregulation. However, the extent to which these signatures are reproducible and transferable across studies varies considerably.

Comparative evaluation of metabolomic investigations suggests that multiple studies report high diagnostic performance using machine learning models, often identifying metabolites associated with central carbon metabolism, amino acid metabolism, and lipid pathways. While these findings are broadly consistent in implicating metabolic reprogramming, the specific metabolites selected as predictive features differ across studies, reflecting variability in cohort characteristics, analytical platforms, and feature selection methods. Studies employing targeted metabolomics approaches tend to report more consistent and biologically interpretable metabolite panels, whereas untargeted approaches identify a broader range of candidate features but with greater variability and reduced reproducibility across datasets.

Similarly, transcriptomic analyses consistently identify oxidative-stress-associated gene signatures linked to immune activation and mitochondrial function. Across studies, genes involved in inflammatory signaling pathways and redox regulation are repeatedly observed; however, the specific gene panels vary and overlap between studies is limited. Network-based approaches such as weighted gene co-expression analysis provide additional insight by identifying regulatory hubs and pathway-level convergence, suggesting that reproducibility may be stronger at the pathway level than at the level of individual genes. Multi-omics integration further enhances this perspective by linking transcriptional, metabolic, and cellular processes, although such approaches are less frequently applied and often lack validation across independent cohorts.

From a computational standpoint, substantial variability exists in model development and evaluation. While many studies report high predictive performance, direct comparison is challenging due to differences in dataset size, class balance, feature dimensionality, and validation strategies. In particular, studies utilizing smaller cohorts and high-dimensional feature spaces tend to report higher performance metrics, raising concerns regarding model overfitting and limited generalizability. In contrast, studies incorporating larger, multi-cohort datasets or independent validation sets generally report more moderate but potentially more reliable performance estimates. The lack of standardized benchmarking frameworks further complicates cross-study comparison and limits the ability to determine the relative robustness of different modeling approaches.

Assessment of methodological quality indicates that variability is most pronounced in the analytical domain, particularly in relation to model validation and reporting transparency. The frequent reliance on internal validation and absence of independent external cohorts limit the ability to assess generalizability and reproducibility of identified biomarkers across diverse populations. In addition, the widespread use of publicly available datasets introduces potential sources of bias related to population heterogeneity, batch effects, and technical variability.

Taken together, the evidence supports the existence of shared oxidative-stress-associated molecular pathways across immune-mediated diseases; however, the reproducibility of specific biomarkers remains variable. Greater consistency is observed at the level of biological pathways and functional networks than at the level of individual molecular features. These findings underscore the importance of integrative and cross-platform approaches, as well as the need for standardized analytical pipelines and rigorous validation frameworks. Strengthening these aspects will be essential to improve comparability across studies and to advance the translation of computationally derived redox signatures into clinically meaningful applications.

## 5. Discussion

The present systematic review integrates current evidence investigating redox-associated mechanisms in immune-mediated diseases through computational analytical approaches. Collectively, the studies included in this review suggest that disruption of redox homeostasis represents a shared molecular feature across several inflammatory disorders, including MS, SLE, LN, RA, SS, and IBD. Despite differences in clinical presentation and organ involvement, these conditions exhibit overlapping mechanisms involving oxidative stress, immune dysregulation, and metabolic perturbations. Recent advances in high throughput molecular profiling combined with computational analysis have enabled a more comprehensive understanding of how redox signaling contributes to immune-mediated disease pathogenesis. The integration of bioinformatics, ML algorithms, and network-based systems biology provides powerful analytical frameworks for identifying molecular signatures associated with oxidative stress and for reconstructing complex regulatory networks that underlie inflammatory pathology.

One of the most consistent findings across the included studies is the presence of metabolic alterations associated with oxidative stress. Metabolomic investigations integrating ML models such as RF, SVM, and ANN demonstrated strong diagnostic performance for distinguishing patients with immune-mediated diseases from healthy individuals. For example, ANN-based analysis of blood metabolomics data achieved high classification accuracy for identifying MS, highlighting the potential of metabolomic signatures for improving diagnostic precision [92].

Similarly, ML-based metabolomic profiling in RA identified significant disruptions in metabolic pathways related to glycolysis and amino acid metabolism, including increased levels of pyruvic acid and phenylalanine and decreased levels of citrulline and carnitine [93]. These metabolic alterations reflect changes in cellular energy metabolism that accompany immune activation and oxidative stress. Additional studies integrating urine and serum metabolomics further demonstrated that ML models can accurately discriminate among multiple immune-mediated diseases using metabolic signatures, supporting the concept that systemic metabolic dysregulation represents a shared feature of inflammatory disorders [94]. Together, these findings emphasize the importance of metabolic reprogramming as a key component of redox-associated immune dysfunction.

Despite these promising findings, several methodological considerations warrant critical evaluation. ML-based approaches for biomarker discovery in OS-associated datasets demonstrate substantial analytical potential; however, their reliability is highly dependent on data structure, model design, and validation rigor. Robust performance is more likely in studies employing adequately powered cohorts, appropriate feature selection strategies, and independent external validation. In contrast, many studies utilize high-dimensional omics datasets with relatively small sample sizes, resulting in an unfavorable feature-to-sample ratio that increases susceptibility to model overfitting and inflated performance estimates. This limitation is particularly relevant in studies reporting very high AUC values, which may not generalize beyond the training dataset. Furthermore, feature stability remains limited, as different studies frequently identify non-overlapping molecular signatures despite converging on similar biological pathways, indicating that reproducibility is greater at the pathway level than at the level of individual biomarkers. Additional sources of variability include batch effects, differences in data generation platforms (e.g., RNA-seq versus microarray; targeted versus untargeted metabolomics), and heterogeneity in preprocessing pipelines, all of which may introduce systematic bias if not rigorously controlled.

Many studies rely predominantly on internal validation frameworks, with limited use of independent external cohorts, thereby restricting assessment of model generalizability. Moreover, ML-derived biomarkers are often not supported by functional or experimental validation, limiting their biological interpretability and translational applicability. Collectively, these considerations highlight that while ML provides powerful tools for identifying OS-associated molecular signatures, careful attention to study design, validation strategy, and biological validation is essential to ensure robustness, reproducibility, and clinical relevance. These limitations highlight that reported predictive performance should be interpreted with caution, particularly in the absence of rigorous validation and reproducibility assessment.

Within this context, and despite these limitations, across multiple immune-mediated diseases, metabolomic investigations integrated with ML algorithms consistently identify metabolic signatures associated with oxidative stress and inflammatory metabolic reprogramming. ML analysis of serum metabolomic profiles in patients with UC identified metabolic signatures associated with disease activity and extent, including perturbations in pathways related to tryptophan–kynurenine metabolism, mitochondrial energy metabolism, amino acid metabolism, and lipid peroxidation processes [97,98]. These metabolic alterations are closely linked to redox imbalance and reflect changes in cellular bioenergetics and inflammatory metabolic reprogramming occurring within intestinal epithelial and immune cell populations during intestinal inflammation.

Importantly, the application of supervised ML algorithms to high dimensional metabolomic datasets enabled identification of predictive metabolic features capable of distinguishing disease states and evaluating treatment response. These findings further reinforce the concept that oxidative stress-associated metabolic remodeling represents a shared molecular feature across multiple ADs and demonstrates the utility of computational metabolomics approaches for identifying disease-associated redox signatures within the intestinal inflammatory microenvironment.

In addition to metabolic alterations, transcriptomic analyses identified several oxidative-stress-related gene networks associated with disease pathogenesis. Computational network approaches such as WGCNA have revealed regulatory genes that function as molecular hubs linking oxidative stress with immune activation. In LN, transcriptomic analysis identified four key genes including *STAT1*, *PRODH*, *TXN2*, and *SETX* that exhibited strong associations with disease activity and immune cell infiltration [82]. Similarly, multi-omics analysis integrating transcriptomics and metabolomics identified several oxidative-stress-related genes including *ABCB1*, *AKR1C3*, *EIF2AK2*, *IFIH1*, *NPC1*, and *SCO2* that demonstrated strong diagnostic performance in SLE [78]. These genes are involved in pathways regulating mitochondrial metabolism, immune signaling, and antiviral responses showing how oxidative stress influences transcriptional programs that contribute to immune-mediated pathology.

Additional transcriptomic investigations integrating ML-based feature selection have further identified oxidative-stress-associated regulatory genes contributing to immune signaling dysregulation in MS. Computational analysis of peripheral blood transcriptomic datasets revealed several hub genes including *MMP9*, *NFKB1*, *NFKBIA*, and *SRC*, which participate in NF-κB–mediated inflammatory signaling pathways and cytokine regulatory networks, providing further evidence that redox-sensitive transcriptional programs contribute to immune activation in neuroinflammatory disease [100].

Glutathione dependent antioxidant pathways also emerged as important regulators of immune function in SLE [52,83,103,104]. The glutathione system plays a critical role in maintaining intracellular redox balance and protecting cells from oxidative damage [105,106]. Integrative analysis combining Mendelian randomization, single cell transcriptomics, and ML modeling identified *LAP3* as a key gene associated with glutathione metabolism and immune dysregulation in SLE [83]. Increased expression of *LAP3* was observed primarily in monocytes and was associated with enhanced inflammatory signaling through the LGALS9 CD44 communication axis. These findings suggest that dysregulation of antioxidant pathways may influence immune cell activation through metabolic mechanisms that promote inflammatory signaling.

Emerging evidence also indicates that oxidative stress contributes to regulated cell death pathways that may play a role in immune-mediated tissue injury. In particular, ferroptosis and cuproptosis have been implicated in LN pathogenesis through mechanisms involving lipid peroxidation and metal dependent oxidative damage. Computational analysis integrating ML algorithms identified *JUN* and *ZFP36* as key regulators of ferroptosis-related cuproptosis pathways in LN [86]. Experimental validation demonstrated increased expression of ferroptosis markers such as transferrin receptor protein 1 and lipid peroxidation products within renal tissue, supporting the role of oxidative-stress-driven cell death in disease progression. These findings highlight a potential mechanistic link between metabolic dysregulation and inflammatory tissue damage. Beyond their value as diagnostic biomarkers, these oxidative-stress-associated transcriptional signatures also provide insight into the regulatory pathways through which redox imbalance influences immune cell signaling and inflammatory responses.

Redox signaling pathways play a critical role in regulating immune cell activation and inflammatory responses in immune-mediated diseases [107,108,109,110,111]. In addition to their cytotoxic effects, ROS function as signaling mediators that modulate intracellular pathways controlling immune cell differentiation, cytokine production, and inflammatory gene expression [35,112,113,114]. Computational analyses integrating transcriptomic and multi-omics datasets have increasingly identified redox-associated signaling networks that influence both innate and adaptive immune responses. For example, systems-level investigation of neuroinflammatory signaling demonstrated that vascular-derived proteins such as fibrinogen can activate microglial inflammatory responses through receptor-mediated pathways that stimulate NADPH oxidase activity and promote ROS-dependent transcriptional programs associated with neuroinflammation [79]. Together, these observations highlight the role of redox-regulated signaling pathways as important modulators of immune activation and inflammatory tissue responses in immune-mediated disease.

Another important insight from the reviewed literature is the value of systems-level approaches for understanding disease heterogeneity. Proteomic and network analysis of SS demonstrated that distinct molecular endotypes correspond to specific clinical symptom clusters [99]. Patients with dryness dominant disease exhibited strong activation of interferon-related pathways and B cell mediated immune responses, whereas individuals with high symptom burden displayed increased expression of inflammatory cytokines and proteins associated with oxidative stress and metabolic regulation. These findings highlight the importance of molecular stratification for understanding disease variability and for guiding the development of targeted therapeutic strategies.

Although these findings highlight the translational potential of computational approaches, a more explicit clinical framework is needed to guide progression toward validation and implementation. In particular, prioritization of candidate biomarkers should emphasize those demonstrating reproducibility across independent cohorts, strong biological relevance to oxidative stress–mediated disease mechanisms, and stability across analytical platforms. Molecular signatures converging on key pathways such as mitochondrial metabolism, glutathione regulation, and inflammatory signaling may represent the most promising candidates for clinical translation. In addition, biomarkers that can be reliably detected in accessible biospecimens, including blood or urine, and that exhibit low intra-individual variability and sustained predictive performance following external validation are more likely to be clinically actionable. Establishing standardized criteria for biomarker selection and validation will be essential to facilitate the transition from computational discovery to clinically relevant diagnostic and prognostic tools.

## 6. Limitations

Several limitations should be considered when interpreting the findings of this systematic review. First, the number of eligible studies identified through the PRISMA screening process was relatively limited. Although the selected studies represented diverse autoimmune conditions and utilized multiple computational approaches, the overall sample size of the literature included in this review remains modest. The relatively small number of studies restricts the ability to draw definitive conclusions regarding the generalizability of computational redox analysis across all immune-mediated diseases. In addition, several disease categories were represented by only a small number of investigations, which may limit the extent to which consistent molecular patterns can be identified across different pathological contexts.

Second, substantial heterogeneity was observed among the included studies with respect to study design, cohort characteristics, data acquisition methods, and analytical approaches. The reviewed studies employed different types of omics datasets including transcriptomics, metabolomics, proteomics, and microbiome profiling. Variability was also present in sequencing platforms, sample preparation protocols, and bioinformatic preprocessing pipelines. In particular, differences across analytical platforms represent an important source of variability. Transcriptomic analyses were performed using both RNA sequencing and microarray-based approaches, which differ in sensitivity, dynamic range, transcriptome coverage, and susceptibility to technical artifacts. RNA sequencing enables detection of low-abundance transcripts and novel transcripts, whereas microarray platforms are limited by predefined probe sets and may be affected by cross-hybridization. Similarly, metabolomic analyses included both targeted and untargeted approaches, which vary in analytical precision, metabolite coverage, and reproducibility. Targeted metabolomics provides accurate quantification of predefined metabolites, while untargeted approaches allow broader metabolite discovery but are more susceptible to batch effects, compound identification challenges, and variability in data processing workflows. These platform-specific differences may influence feature detection, downstream analyses, and the identification of molecular signatures, thereby complicating direct comparison across studies. Furthermore, the machine learning models used across studies differed in algorithm selection, training procedures, and validation strategies, which may affect the reported predictive performance of diagnostic models. Such methodological variability may limit reproducibility and highlight the need for greater standardization in data acquisition, preprocessing, normalization, and cross-platform validation to improve consistency across studies.

Third, many of the included investigations relied on publicly available datasets obtained from repositories such as the Gene Expression Omnibus or other open access databases. While these resources provide valuable data for exploratory analyses, they often originate from independent cohorts with differing demographic characteristics, clinical inclusion criteria, and experimental conditions. These factors may introduce variability and potential confounding effects that are difficult to control within secondary analyses. In addition, several studies lacked extensive external validation cohorts, which limits the assessment of whether identified biomarkers maintain predictive accuracy across independent populations.

Another important limitation relates to the inherent constraints of computational inference. Although machine learning and systems biology approaches are powerful tools for identifying molecular associations and regulatory networks, these methods primarily generate predictive or correlative findings rather than direct evidence of causality. Many of the candidate genes, metabolites, and signaling pathways identified in the reviewed studies therefore require experimental validation through functional assays and mechanistic investigations. Without such validation, the biological significance of computationally identified biomarkers remains partially speculative.

In addition to these considerations, important methodological limitations related to computational modeling warrant further attention. Although several studies reported high predictive performance, including area under the curve values ≥0.95, such findings should be interpreted cautiously given the risk of overfitting, particularly in the context of high-dimensional omics data and limited sample sizes. In some cases, model development involved extensive feature selection without implementation of robust validation strategies, such as nested cross-validation or appropriately regularized modeling approaches, increasing the likelihood of optimistic performance estimates. Furthermore, reliance on internal validation procedures without independent external validation cohorts limits the ability to assess generalizability across diverse populations. The frequent use of publicly available datasets introduces additional challenges related to population heterogeneity, batch effects, and technical variability, which may further compromise model transferability. Addressing these issues through standardized reporting practices, rigorous validation frameworks, and inclusion of independent, multi-center datasets will be essential to improve reproducibility and support the clinical translation of computational models in this field.

Despite these limitations, the studies included in this systematic review collectively provide important insights into how computational approaches can be used to investigate redox-associated mechanisms in immune-mediated diseases. Continued integration of larger multi-omics datasets, standardized analytical frameworks, and experimental validation studies will be necessary to strengthen the reliability and clinical applicability of computational redox analysis.

## 7. Future Directions

Future research should focus on expanding the integration of multi-omics datasets with advanced computational methods in order to further clarify the role of redox-associated pathways in immune-mediated diseases. Although the studies included in this review demonstrate the potential of computational analysis for identifying redox-related biomarkers, many investigations rely on relatively small datasets or publicly available repositories. Future studies incorporating larger patient cohorts, longitudinal sampling, and multi center datasets will be essential to improve the robustness and reproducibility of computational findings. Longitudinal analyses may be particularly valuable for understanding how oxidative stress-related molecular signatures evolve throughout disease progression and in response to therapeutic interventions.

Another important direction for future research involves improving the interpretability and clinical applicability of machine learning models. Although ML algorithms such as RF, SVM, and ANN have demonstrated strong diagnostic performance in several studies, the clinical translation of these models requires transparent and reproducible analytical frameworks. The increasing application of explainable artificial intelligence approaches may help address this challenge by providing interpretable models that allow researchers and clinicians to understand the contribution of individual molecular features to predictive outcomes. Integration of explainability methods with multi-omics datasets may also facilitate the identification of causal pathways linking oxidative stress with immune dysregulation.

Future investigations should also prioritize experimental validation of computationally identified biomarkers and regulatory networks. While computational approaches are effective for identifying candidate genes, metabolites, and signaling pathways associated with disease, functional studies are necessary to confirm the biological relevance of these findings. Experimental models examining redox-associated pathways such as glutathione metabolism, ferroptosis, and cuproptosis may provide further insight into how metabolic and oxidative processes contribute to immune-mediated tissue injury. Such mechanistic investigations will be essential for translating computational discoveries into therapeutic strategies.

In addition, future research should explore the potential for integrating redox-related biomarkers into clinical decision-making frameworks. Computational analysis of redox-associated molecular signatures may enable the development of personalized diagnostic tools capable of stratifying patients based on disease mechanisms rather than clinical symptoms alone. This approach may also support the identification of patient subgroups that are more likely to respond to targeted therapeutic interventions aimed at restoring redox homeostasis or modulating metabolic pathways involved in immune activation.

## 8. Conclusions

This systematic review highlights the growing importance of computational approaches for investigating redox-associated mechanisms in immune-mediated diseases. Across multiple disorders including MS, SLE, LN, RA, SS, and IBD, the reviewed studies consistently demonstrate that oxidative stress plays a central role in immune dysregulation, metabolic reprogramming, and inflammatory tissue injury. Advances in high throughput molecular technologies combined with computational analysis have enabled the identification of complex molecular signatures linking oxidative stress with disease pathogenesis.

**Figure 5:**
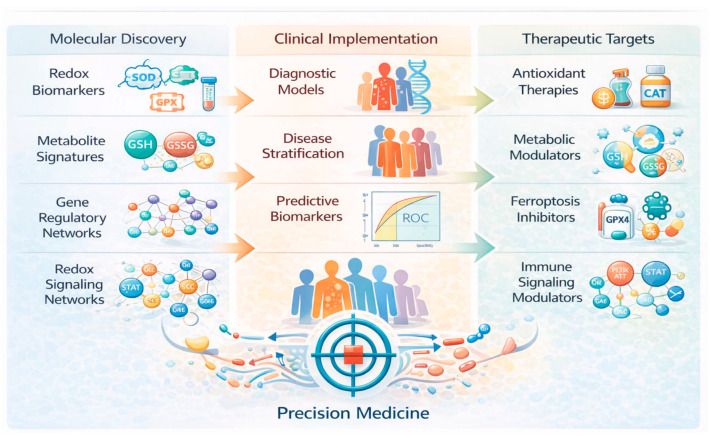
Translational framework of redox biology in precision medicine. Computational and multi-omics analyses enable the identification of oxidative-stress-associated molecular features, including redox biomarkers, metabolite signatures, gene regulatory networks, and redox signaling pathways. These molecular discoveries support clinical implementation through the development of diagnostic models, disease stratification strategies, and predictive biomarkers derived from machine learning and bioinformatic analyses. Integration of these approaches facilitates the identification of therapeutic targets, including antioxidant therapies, metabolic modulators, ferroptosis inhibitors, and immune signaling modulators. Created in BioRender. Mittal, R. (2026) https://BioRender.com/0ceg71e (accessed on 19 April 2026) .

Machine learning models have demonstrated strong diagnostic performance when applied to metabolomic and transcriptomic datasets, suggesting that redox-associated molecular features may serve as valuable biomarkers for disease detection and classification. The translational framework linking redox-based molecular discovery with clinical implementation and therapeutic targeting is summarized in Figure 5. Systems biology approaches have further revealed regulatory networks that connect oxidative stress with immune signaling pathways and metabolic processes. In addition, emerging evidence suggests that oxidative stress contributes to regulated cell death pathways such as ferroptosis and cuproptosis, providing new insight into mechanisms of tissue injury in inflammatory diseases.

Overall, the integration of computational analysis with multi-omics datasets provides a powerful framework for advancing the understanding of redox biology in immune-mediated diseases. Continued development of computational redox analysis has the potential to improve biomarker discovery, enhance disease stratification, and support the development of targeted therapeutic strategies aimed at restoring redox balance and immune homeostasis.

## Figures and Tables

**Table 1:** A summary of included studies.

Study	Disease	Data	Computational Method	Key Biomarkers/Features	Main Findings
Zhang et al. [86]	LN	Transcriptomics	LASSO, RF, SVM-RFE	JUN, ZFP36	• Identified ferroptosis/cuproptosis-related genes in LN. • Reduced JUN and ZFP36 associated with immune infiltration. • Diagnostic model showed high predictive accuracy.
Zhou et al. [78]	SLE	Multi-omics (Tx + metabolomics)	RF, LASSO, DL	ABCB1, AKR1C3, EIF2AK2, IFIH1, NPC1, SCO2	• Identified OS-related gene signature in SLE. • Metabolic alterations associated with mitochondrial respiration. • SCO2 and EIF2AK2 showed strong diagnostic accuracy (AUC > 0.96).
Mendiola et al. [79]	MS models	Multi-omics (Tx + proteomics)	Network analysis	ITGAM, fibrinogen	• Fibrinogen identified as driver of microglial activation. • MAPK1/MAPK3 signaling and NADPH oxidase increased ROS production. • Disruption of fibrinogen–ITGAM signaling reduced neuroinflammatory responses.
Zeng et al. [82]	LN	Transcriptomics	WGCNA, LASSO	STAT1, PRODH, TXN2, SETX	• Identified OS-associated gene module linked to renal inflammation. • STAT1 and SETX upregulated in LN. • Gene model predicted disease with AUC ≈0.93.
Wang et al. [83]	SLE	Multi-omics + scRNA-seq	CatBoost, XGBoost, LASSO	LAP3	• Glutathione metabolism identified as key redox pathway. • LAP3 expression associated with inflammatory monocytes. • Gene showed strong diagnostic performance.
Ata et al. [92]	MS	Blood metabolomics	ANN	Metabolic profile features	• ANN classified MS vs HC with ~87% accuracy. • Metabolite alterations linked to mitochondrial metabolism. • Demonstrates potential for noninvasive diagnostic modeling.
Yagin et al. [93]	RA	Plasma metabolomics	EBM	Pyruvic acid, phenylalanine	• ML model identified nonlinear metabolic thresholds. • Metabolites linked to glycolysis and OS pathways. • Diagnostic model achieved AUC ≈ 0.90.
Du et al. [94]	AD	Urine + serum metabolomics	NB, RF, NN	Amino acid and lipid metabolites	• ML models classified multiple AD with high accuracy. • Urine metabolomics showed better performance than serum. • Identified shared metabolic dysregulation across AD.
Lei et al. [95]	IBD	Urine metabolomics	RF, SVM	Xylose, L-fucose, citric acid	• Urinary metabolite panel differentiates UC and CD. • Dysregulation observed in glycolysis and TCA cycle metabolites. • ML models maintained accuracy across disease severity.
Ning et al. [96]	IBD	Multi-omics (microbiome + metabolomics)	RF integration	Microbial species panel, metabolite panel	• Identified 74 altered microbial species in IBD. • Integrated multi-omics model achieved AUROC up to 0.98. • Demonstrated interaction between microbial dysbiosis and host metabolism.
Ge et al. [97]	UC	Serum metabolomics	RF, LASSO, SVM-RFE	Tridecanoic acid, pelargonic acid	• Serum metabolite signatures distinguish UC from HC. • Tridecanoic acid associated with extensive disease. • ML models outperformed conventional inflammatory biomarkers.
Han et al. [98]	UC	Plasma metabolomics	RF, SVM	12-hydroxydodecanoic acid, phenylacetaldehyde, AMP	• Identified plasma metabolite signature predicting Tx escalation. • Altered PC metabolism associated with disease progression. • RF achieved highest predictive performance (AUC ≈ 0.97).
Berry et al. [99]	SS	Proteomics	ARACNE	IL6, APEX1, TIGAR	• Identified molecular subtypes of SS. • High symptom burden subtype associated with OS markers. • IL6 pathway suggested as therapeutic target.
Ma et al. [100]	MS	Transcriptomics	PPI network, LASSO	MMP9, NFKB1, NFKBIA, SRC	• Identified OS-associated gene signature in MS. • Genes linked to NF-κB signaling and immune activation. • Predictive model demonstrated strong performance.
Wu et al. [101]	MS (EAE model)	RNA-seq	LASSO, ENet, RF	Ngp, CD180, F10	• Identified microglial gene signature in neuroinflammation. • Genes enriched in TLR and IL17 signaling pathways. • ML models demonstrated strong diagnostic capability.
Wang et al. [102]	RA, MS, T1D	Multi-omics + scRNA-seq	LASSO, GSEA	ROMO1	• ROMO1 identified as shared OS regulator. • Associated with mitochondrial ROS production. • Expression varies across immune cell populations.

AD—autoimmune disease; AMP—4-aminophenol; ANN—artificial neural network; ARACNE—algorithm for the reconstruction of accurate cellular networks; AUC—area under the curve; CD—Crohn’s disease; DL—deep learning; EBM—explainable boosting machine; EAE—experimental autoimmune encephalomyelitis; ENet—elastic net; GSEA—gene set enrichment analysis; HC—healthy controls; IBD—inflammatory bowel disease; LASSO—least absolute shrinkage and selection operator; LN—lupus nephritis; ML—machine learning; MS—multiple sclerosis; NB—naïve Bayes; NN—neural network; OS—oxidative stress; PC—phosphatidylcholine; PPI—protein–protein interaction; RA—rheumatoid arthritis; RF—random forest; ROSs—reactive oxygen species; scRNA-seq—single-cell RNA sequencing; SLE—systemic lupus erythematosus; SS—Sjögren’s syndrome; SVM—support vector machine; TCA—tricarboxylic acid; TLR—Toll-like receptor; Tx—treatment; UC—ulcerative colitis; WGCNA—weighted gene co-expression network analysis.

**Table 2:** Cross-study synthesis of computational findings and methodological patterns.

Analytical Dimension	Cross-Study Observation	Consistency Across Studies	Key Sources of Variability	Implications for Interpretation	References
Biological Pathways Identified	Recurrent involvement of mitochondrial dysfunction, inflammatory signaling, glutathione metabolism, and metabolic reprogramming	High (pathway-level convergence across diseases)	Disease context, omics modality	Supports shared redox-driven mechanisms across immune-mediated diseases	Zhou et al., 2025; Zeng et al., 2024; Wang et al., 2025a; Ge et al., 2025; Han et al., 2025 [78,82,83,97,98]
Molecular Feature Reproducibility	Limited overlap of specific genes/metabolites across studies	Low (feature-level inconsistency)	Platform differences, cohort composition, feature selection methods	Biomarker reproducibility is weaker at individual feature level	Zhou et al., 2025; Zeng et al., 2024; Yagin et al., 2025; Du et al., 2023 [78,82,93,94]
Metabolomic vs. Transcriptomic Findings	Metabolomics consistently identifies metabolic dysregulation; transcriptomics highlights immune and signaling pathways	Moderate–high (complementary consistency)	RNA-seq vs. microarray; targeted vs. untargeted metabolomics	Multi-omics integration improves biological interpretation	Zhou et al., 2025; Zeng et al., 2024; Yagin et al., 2025; Ge et al., 2025 [78,82,93,97]
Machine Learning Performance	Many studies report high diagnostic performance (AUC often >0.90)	Moderate (performance varies by dataset and method)	Sample size, feature dimensionality, validation strategy	High performance may not reflect real-world generalizability	Zhou et al., 2025; Du et al., 2023; Ning et al., 2023; Han et al., 2025 [78,94,96,98]
Validation Approaches	Predominant use of internal validation methods	Low consistency in rigorous validation	Limited external cohorts, dataset reuse	Limits assessment of reproducibility and clinical applicability	Ata et al., 2024; Yagin et al., 2025; Du et al., 2023; Han et al., 2025 [92,93,94,98]
Data Sources	Frequent reliance on publicly available datasets	High (common practice across studies)	Population heterogeneity, batch effects	Introduces bias and limits cross-study comparability	Zhou et al., 2025; Zeng et al., 2024; Ning et al., 2023 [78,82,96]
Computational Approaches	Broad use of ML (RF, SVM, ANN) and network-based methods	High (methodological convergence)	Differences in preprocessing, feature selection, tuning	Lack of standardization affects reproducibility	Zhou et al., 2025; Zeng et al., 2024; Ata et al., 2024; Yagin et al., 2025; Ning et al., 2023 [78,82,92,93,96]
Systems-Level Insights	Strong convergence at pathway and network level rather than individual biomarkers	High (network-level consistency)	Study design, integration depth	Suggests pathway-level biomarkers may be more robust than single features	Wang et al., 2025a; Berry et al., 2025 [83,99]

## Data Availability

No new data were created or analyzed in this study. Data sharing is not applicable to this article.

## Supplementary Materials

The following supporting information can be downloaded at: https://www.mdpi.com/article/10.3390/antiox15050548/s1, Table S1. A comparative summary of datasets, validation strategies, and key findings across included studies.

## Glossary of Computational and Machine Learning Terms

To facilitate interpretation of computational methodologies, key ML and systems biology terms used in this review are briefly defined below:

ANN (Artificial Neural Network)	A computational model inspired by neural systems, capable of capturing complex nonlinear relationships within high-dimensional datasets.
ENet (Elastic Net)	A regularized regression method that combines L1 and L2 penalties to improve feature selection and model stability, particularly in correlated high-dimensional data.
GSEA (Gene Set Enrichment Analysis)	A statistical method used to determine whether predefined sets of genes show significant differences between biological states.
L1 Regularization	A method that applies a penalty equal to the absolute values of model coefficients, promoting sparsity and enabling feature selection in high-dimensional datasets.
L2 Regularization	A method that applies a penalty based on the squared magnitude of model coefficients, reducing overfitting by shrinking coefficients without eliminating features.
LASSO (Least Absolute Shrinkage and Selection Operator)	A regression-based method that performs feature selection by applying L1 regularization, enabling identification of the most informative variables in high-dimensional datasets.
PPI (Protein–Protein Interaction Network)	A network representation of physical or functional interactions between proteins, used to identify key regulatory hubs.
RF (Random Forest)	An ensemble learning method that constructs multiple decision trees and aggregates their outputs to improve classification accuracy and reduce overfitting.
SHAP (SHapley Additive exPlanations)	A model interpretation method that quantifies the contribution of individual features to model predictions.
SVM (Support Vector Machine)	A supervised learning algorithm that identifies optimal hyperplanes to separate data into distinct classes.
WGCNA (Weighted Gene Co-expression Network Analysis)	A network-based approach used to identify clusters of co-expressed genes and detect modules associated with biological traits or disease phenotypes.
