# Supplementary material for: Oxidative-Stress-Associated Molecular Signatures in Immune-Mediated Diseases: A Systematic Review Integrating Machine Learning and Systems Biology Approaches
Source: Antioxidants (Basel). 2026 Apr 26;15(5):548. doi: 10.3390/antiox15050548 (PMC13203210; doi:10.3390/antiox15050548)
Supplement: Supplementary file 1 [file antioxidants-15-00548-s001.zip › antioxidants-4235293-supplementary.pdf]

Supplementary Table S1. A comparative summary of datasets, validation strategies, and key findings across included studies

Study (Author, Year)	Disease	Dataset Type	Sample Size*	Validation Cohort	ML / Computational Models	Key Oxidative Stress Pathways Identified
Han et al., 2025	UC	Plasma metabolomics (untargeted)	Not consistently reported	No (internal validation)	RF, SVM	Lipid metabolism, mitochondrial metabolism, oxidative stress–related metabolic pathways
Ge et al., 2025	UC	Serum metabolomics	Not consistently reported	No (internal validation)	RF, SVM, LASSO	Tryptophan–kynurenine metabolism, lipid peroxidation, mitochondrial metabolism
Lei et al., 2025	IBD	Urine metabolomics (targeted)	Not consistently reported	No (internal validation)	RF, SVM	Glycolysis, TCA cycle, microbial metabolism
Ning et al., 2023	IBD	Multi-omics (microbiome + metabolomics)	>1300 (multi-cohort)	Yes (independent cohort)	RF integration	Amino acid metabolism, host–microbiome metabolic interaction, redox pathways
Ata et al., 2024	MS	Blood metabolomics	756 (515 MS, 241 HC)	Internal split only	ANN	Mitochondrial metabolism, redox balance
Yagin et al., 2025	RA	Plasma metabolomics	Not consistently reported	Internal validation	Explainable Boosting Machine	Glycolysis, mitochondrial metabolism, oxidative stress pathways
Du et al., 2023	Multiple AD	Urine + serum metabolomics	267	Internal validation	NB, RF, NN	Amino acid metabolism, lipid metabolism, central carbon metabolism
Zeng et al., 2024	LN	Transcriptomics	Not consistently reported	Limited validation dataset	WGCNA, LASSO	JAK–STAT signaling, PI3K–Akt, oxidative stress response
Zhou et al., 2025	SLE	Multi-omics (Tx + metabolomics)	Not consistently reported	Limited validation	RF, LASSO, DL	Mitochondrial respiration, oxidative phosphorylation, redox metabolism

**Study (Author, Year)**	**Disease**	**Dataset Type**	**Sample Size***	**Validation Cohort**	**ML / Computational Models**	**Key Oxidative Stress Pathways Identified**
Wang et al., 2025a	SLE	Multi-omics + scRNA-seq	Not consistently reported	Limited validation	CatBoost, XGBoost, LASSO	Glutathione metabolism, immune signaling
Ma et al., 2025	MS	Transcriptomics	Not consistently reported	Internal validation	LASSO, PPI network	NF-κB signaling, inflammatory pathways
Berry et al., 2025	SS	Proteomics	Not consistently reported	No formal validation	ARACNE	Inflammatory signaling, oxidative stress response
Wang et al., 2025b	RA, MS, T1D	Multi-omics + scRNA-seq	Not consistently reported	Limited validation	LASSO, GSEA	Mitochondrial ROS production, redox regulation
Zhang et al., 2024	LN	Transcriptomics	Not consistently reported	Internal validation	RF, SVM-RFE, LASSO	Ferroptosis, cuproptosis, lipid peroxidation
Wu et al., 2025	MS (EAE)	RNA-seq	Not consistently reported	Internal validation	RF, ENet, LASSO	TLR signaling, IL-17 pathway, inflammatory signaling
Mendiola et al., 2024	MS models	Multi-omics (Tx + proteomics)	Not consistently reported	No formal validation	Network analysis	MAPK signaling, NADPH oxidase, ROS production

Abbreviations

AD – autoimmune disease; AMP – 4-aminophenol; ANN – artificial neural network; ARACNE – algorithm for the reconstruction of accurate cellular networks; AUC – area under the curve; CD – Crohn's disease; DL – deep learning; EBM – explainable boosting machine; EAE – experimental autoimmune encephalomyelitis; ENet – elastic net; GSEA – gene set enrichment analysis; HC – healthy controls; IBD – inflammatory bowel disease; LASSO – least absolute shrinkage and selection operator; LN – lupus nephritis; ML – machine learning; MS – multiple sclerosis; NB – naïve Bayes; NN – neural network; OS – oxidative stress; PPI – protein–protein interaction; RA – rheumatoid arthritis; RF – random forest; ROS – reactive oxygen species; scRNA-seq – single-cell RNA sequencing; SLE – systemic lupus erythematosus; SS – Sjögren's syndrome; SVM – support vector machine; TCA – tricarboxylic acid; TLR – Toll-like receptor; Tx – treatment; UC – ulcerative colitis; WGCNA – weighted gene co-expression network analysis.
